# Exploring the Maximum Detectable Percentage of Choriocapillaris Flow Deficits Within Geographic Atrophy Using SS-OCT Angiography

**DOI:** 10.1167/iovs.67.11.20

**Published:** 2026-09-11

**Authors:** Sara Beqiri, Marjan Imani Fooladi, Niccolò Ascioti, Bhagavath S. Kumar, Hefu Pan, Mengxi Shen, Alessandro Berni, Gissel Herrera, Omar S. El-Mulki, Omar Badla, Winston Lam, Eduardo Herrera, Omer Trivizki, Maura Di Nicola, Nadia K. Waheed, Robert C. O'Brien, Ruikang K. Wang, Giovanni Gregori, Philip J. Rosenfeld

**Affiliations:** 1 Department of Ophthalmology, Bascom Palmer Eye Institute, University of Miami Miller School of Medicine, Miami, Florida, United States; 2 Department of Bioengineering, University of Washington, Seattle, Washington, United States; 3 Department of Ophthalmology, IRCCS San Raffaele Scientific Institute, Milan, Italy; 4 Department of Ophthalmology, Tel Aviv Medical Center, Tel Aviv, Israel; 5 New England Eye Center, Tufts Medical Center, Tufts University School of Medicine, Boston, Massachusetts, United States; 6 Department of Ophthalmology, University of Washington, Seattle, Washington, United States

**Keywords:** swept-source OCT angiography (SS-OCTA), age-related macular degeneration (AMD), choriocapillaris flow deficits (CCFDs), geographic atrophy (GA)

## Abstract

**Purpose:**

Swept-source optical coherence tomography angiography (SS-OCTA) was used to longitudinally image eyes with AMD to determine the maximum percentage of choriocapillaris flow deficits (CCFD%) within geographic atrophy (GA).

**Methods:**

A retrospective review was performed of prospectively collected SS-OCTA images of eyes with AMD and at least one large hypertransmission defect (hyperTD). Eyes were followed up for five years, by which time unambiguous GA had developed. A grid registration strategy was used to measure the change of CCFD% at the center of atrophy and in the surrounding regions.

**Results:**

Twenty eyes were analyzed over six visits separated by 12 ± 3 months. The mean hyperTD area across 20 eyes increased from 2.90 mm^2^ at visit 1 to 8.59 mm^2^ by visit 6 (*P* < 0.001), whereas the CCFD% at the target box placed at the center of atrophy showed no statistically significant change with a maximum recorded value of 43.8% by the final visit. Non-target boxes were subdivided into boxes affected by hyperTDs (hyperTD boxes) and boxes unaffected by hyperTDs at any timepoint (non-hyperTD boxes). A statistically significant CCFD% difference was noted between boxes, with the hyperTD boxes showing increases over time.

**Conclusions:**

CCFDs% measurements within atrophy never showed the complete loss of CC at the center of GA. This phenomenon is likely due to the displacement of choroidal vessels into the CC slab where their angiographic signal is detected.

Geographic atrophy (GA), the late-stage of nonexudative AMD, was historically diagnosed using color fundus imaging or fundus autofluorescence imaging, but optical coherence tomography (OCT) imaging has become the new gold-standard for imaging GA.^1^^–^^3^ On OCT imaging, GA appears as complete retinal pigment epithelium and outer retinal atrophy^3^ and is associated with the loss of the outer retina, RPE and choriocapillaris (CC).^4^^,^^5^ One of the advantages of using OCT to diagnose GA is that the same scan pattern can be used to identify OCT structural biomarkers that predict the onset and progression from intermediate to late-stage AMD.^6^^–^^8^ These high-risk OCT biomarkers include drusen area and volume, the area of hyperreflective foci (HRF), and the presence of calcified drusen (CaD), which predict the onset of large hypertransmission defects (hyperTDs), the earliest persistent change that eventually progresses to GA.^6^^–^^8^ Large hyperTDs are defined by using en face OCT imaging of dense raster OCT volume scans to identify bright lesions with a greatest linear dimension (GLD) of at least 250 µm.^9^^–^^13^ Once a hyperTD reaches a GLD of 250 µm and is categorized as a large hyperTD, it shows a persistence rate of 99.6%, and will eventually progress to GA, therefore we consider the term large hyperTD as synonymous to GA.^12^

We prefer the use of swept-source OCT angiographic (SS-OCTA) scans to define these high-risk OCT biomarkers because we can use the same scans to image the underlying CC.^14^^–^^22^ SS-OCTA can non-invasively obtain an angiographic signal by using algorithms such as the optical microangiographic algorithm (OMAG) that detects the motion of red blood cells by using the flow decorrelation signal between at least two B-scans repeated at the same location.^23^ Through a series of guideline articles, we refined our strategy for imaging and quantifying the CC by selecting an anatomically appropriate slab combined with thresholding, binarizing, and compensating the angiographic signal that address the artifacts associated with measuring CC flow deficits (CCFDs).^14^^,^^15^^,^^21^^,^^24^^–^^28^

By combining SS-OCTA imaging with our CCFD quantification strategies, we have investigated the role of CC perfusion in the formation of large hyperTDs as a biomarker of AMD onset and progression.^22^^,^^29^ Using longitudinal natural history SS-OCTA imaging on iAMD patients, we have shown that CC flow deficits do not appear to increase before the formation of large hyperTDs, which was contrary to longstanding beliefs about the role of CC perfusion as a cause of hyperTD formation.^22^ Our results are consistent with the recently reported results from Hwang et al.^29^ that showed a decrease in CC blood flow velocity in the area of a hyperTD only after the formation and enlargement of hyperTDs. These findings of CC loss and decreased perfusion after the formation of large hyperTDs are consistent with our previous findings that CC flow deficits increased around established GA and the increase in these CCFDs around GA and throughout the entire scan area correlated with the enlargement rate of GA.^16^^,^^18^ The totality of the current evidence involving CC perfusion of large hyperTDs (GA) suggests that the formation of hyperTDs is independent of focal CC perfusion deficits, while the enlargement rate of these large hyperTDs is dependent on CC perfusion throughout the macula. Although our previous reports explored the correlation between localized and global macular CC flow impairment around GA with the growth rate of GA, we never explored the CC perfusion within the area of GA or large hyperTDs. In this present study, we investigated the changes in CC perfusion within GA as the GA enlarged. We found that the loss of the CC within the GA region results in the anterior displacement of the larger choroidal vessels within the area of atrophy that resulted in a ceiling effect when detecting CCFD values that prevents us from measuring the complete loss of CC perfusion.

## Methods

Patients diagnosed with AMD were enrolled in an ongoing prospective observational SS-OCTA imaging study at the Bascom Palmer Eye Institute. This study was approved by the University of Miami Miller School of Medicine Institutional Review Board, and all participants signed an informed consent. The study was conducted in alignment with the Declaration of Helsinki and the Health Insurance Portability and Accountability Act of 1996.

### Participants

A retrospective review of SS-OCTA images of AMD subjects in our study identified eyes with hyperTDs and a minimum of six annual follow-up visits with visits at 12- ± 3-month intervals. All eyes with pre-existing exudative MNV, retinal pigment epithelial tears, and other retinal diseases such as diabetic retinopathy, vascular occlusions, and significant vitreoretinal interface diseases such as advanced epiretinal membranes causing distortion of the retina, vitreomacular traction, or previous vitreoretinal surgery were excluded.

### Imaging Protocol

All patients underwent fovea-centered 6 × 6 mm SS-OCTA imaging (PLEX Elite 9000; Carl Zeiss Meditec, Dublin, CA, USA), acquired by one of two trained imaging technicians. The SS-OCTA device uses a swept-source laser with a central wavelength of 1050 nm and bandwidth of 100 nm. Our 6 × 6 mm imaging protocol utilized a scanning speed of 100,000 A-scans per second, acquiring 500 A-scans per B-scan and 500 B-scans along the 6 mm slow axis with a uniform 12 µm spacing between both A-scans and B-scans. Each B-scan was repeated twice at the same location to obtain angiographic signal information that was processed using the instrument's complex optical microangiographic algorithm. Scans were reviewed for signal strength and image quality, and excluded if signal was below 7 or significant motion artefacts were present. If several scans were available for the same visit, the highest quality scan was selected.

### Masking and Compensation Strategy

We followed our most updated masking strategy to minimize all potential sources of artifacts when performing CCFD% measurements.^22^^,^^27^^,^^28^ Choroidal hypotransmission defects (hypoTDs) appear as dark foci on the en face subretinal pigment epithelium (subRPE) image. These hypoTDs correspond to shadows under calcified drusen (CaD) or hyperreflective foci (HRF), which obstruct light penetration into the choroid.^6^^–^^8^^,^^30^ As the CC cannot be reliably quantified in these regions, we created an exclusion mask to remove all areas of hypoTDs from further analysis and quantification.^28^^,^^31^^,^^32^

Choroidal hyperTDs appear as bright areas on the same en face subRPE slab used to identify hypoTDs.^9^^,^^11^^,^^12^^,^^33^^,^^34^ They correspond to areas of increased light penetration into the choroid due to RPE and outer retinal attenuation. As demonstrated by Berni et al.,^28^ applying compensation over these hyperTDs will generate artifactual CCFDs, therefore we created a compensation mask to avoid compensating these regions.

To reduce the impact of light attenuation under regions of drusen, we applied the compensation strategy described by Berni et al.^28^ and more recently implemented by Beqiri et al.^22^ Briefly, we considered a range of compensation levels on the CC structure slab, characterized by a compensation parameter gamma (γ). We then determined the optimal gamma, which is the value resulting in the most homogeneous OCT signal across the CC structure slab, and this value was applied to compensate the CC flow image.

### CCFD Binarization and Quantification

A semi-automated algorithm was used to produce a 16 µm thick CC slab positioned at 4 µm to 20 µm under the Bruch's membrane (BM). This slab essentially corresponds to the anatomical location of the CC, and its use for the quantification of the CC has been validated by Chu et al.^27^ The segmentation output was then reviewed and manually corrected within the software wherever necessary.

**Figure 1.**
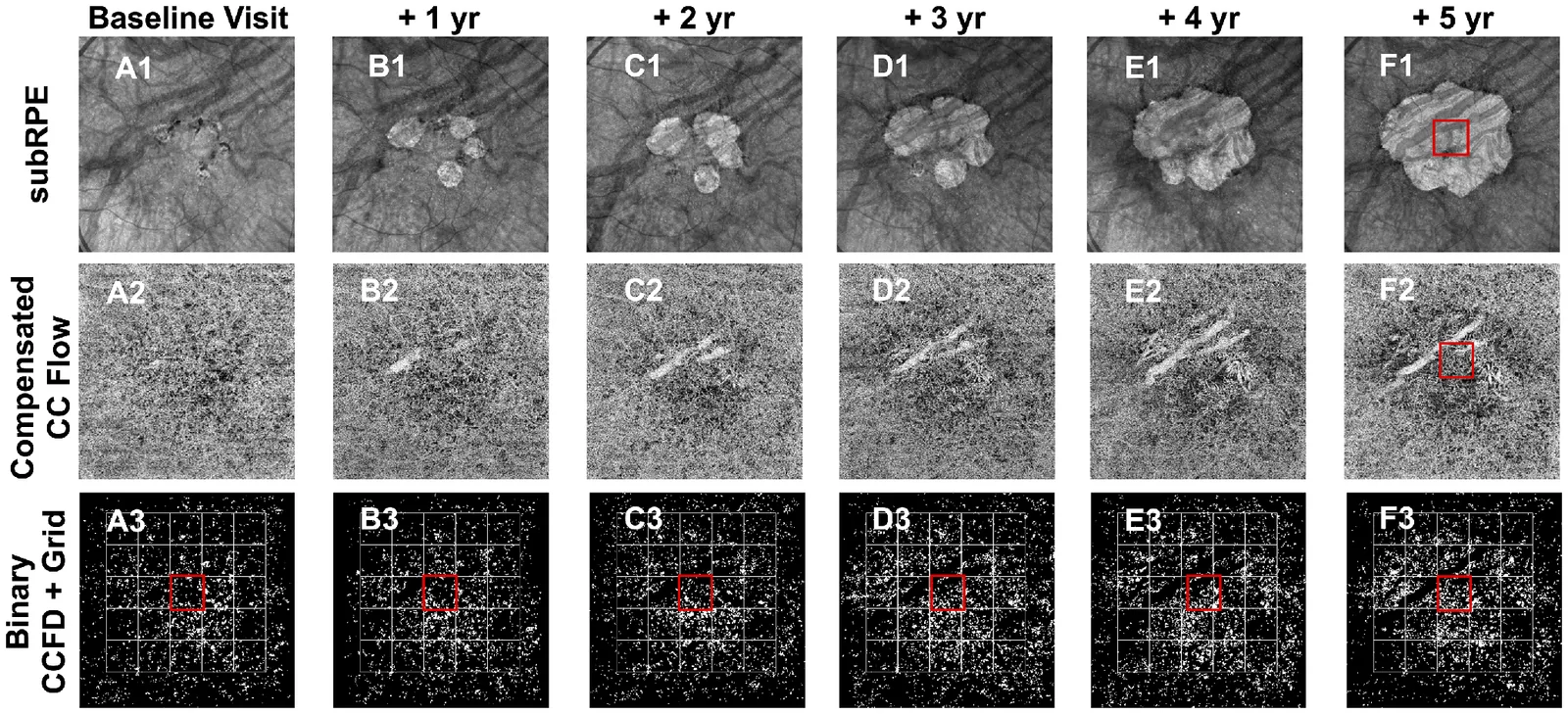
Example of an eye with AMD showing the onset and progression of choroidal hypertransmission defects (hyperTD). **(A1–F1)** The en face SS-OCTA subRPE slab across six visits over five years. HyperTDs are seen as bright areas on the subRPE slab and their size grew from 0.08 mm^2^ at baseline **(A1)** to 8.47 mm^2^ at the final visit **(F1)**. **(F1)** The location of the red target box at the center of the hyperTD in the final visit. **(A2–F2)** The compensated CC flow images. Loss of CC corresponds to dark areas on the *en face* images, and the underlying choroidal vessels become more prominent as the hyperTD enlarges. Panel F2 shows the location of the target box in red. **(A3–F3)** The binary CCFD maps obtained by thresholding the images in **A2–F2**. *White areas* on the binary maps represent flow deficits. All binary maps are registered to the final visit and the superimposed grid is used to track the CCFDs measurements from the same regions over time. The *red box* highlights the location of the target box selected at a location in the center of atrophy. In this example case, the target box CCFD% values range from 13.6% in the baseline visit **(A3)** to 25.5% in the final visit **(F3)**.

After applying the masks and compensating the CC images, a Fuzzy C-means global thresholding algorithm was used to binarize the FD maps. All perfusion deficits with a maximal linear dimension below 24 µm, which corresponded to the physiological intercapillary distance, were removed because these smaller deficits are considered to represent noise.^14^^,^^21^^,^^24^^,^^25^^,^^27^^,^^35^^,^^36^ The output was a binary map where flow deficits are shown as white areas positioned on a black background of detectable flow (Fig. 1, Panels A3–F3).

### Grid Registration

We implemented a grid strategy that was previously described^21^^,^^22^ to track localized CCFD% changes across visits. The images of a given eye at different visits were all registered to the one at the final visit. This registration was also applied to all exclusion masks, which were then combined into one “integrated” mask to ensure that the same regions were removed from the analysis of CCFD% measurements over time.

The target region was selected at the center of the hyperTD identified at the final visit for each eye by manually placing a 74 × 74 pixel (approx. 0.9 × 0.9 mm) box so that it would be fully surrounded by atrophic RPE on all four borders (Fig. 1F1). Therefore only eyes in which the total area of the hyperTD exceeded the size of a square box (0.81 mm^2^) at the final visit of the selected six-visit series were included. This box size was chosen to match the grid strategy described by Hiya et al.,^21^ who performed a repeatability analysis to determine the minimal detectable change thresholds for true CCFD% change versus test-retest variability. Conforming to the same box dimensions allows standardization with their previously established values.

Our grid registration algorithm allowed us to position the target box at the center of the hyperTD and the algorithm automatically fit a grid of equally sized boxes around the target box that could be fully contained within the scan region and the CCFD map. This grid was positioned on all the registered images allowing the same regions to be consistently tracked and compared throughout all visits (Figs. 1A3–F3). The grid had to be contained within each of the scans so that there were no boxes encroaching the edges of any scan. Any grid box where more than 25% of its area could not be quantified either due to the exclusion mask or pixel intensity at 5 dB below the median background signal, was excluded from the analysis as previously described.^22^ If this criterion applied to the target box, then the entire case would be excluded.

### Statistical Methods

Generalized estimating equations with robust standard errors for clustered data were used to analyze CCFD% and hyperTD across visits.^37^^–^^39^ Tukey's method was used to adjust for multiple comparisons. The cluster bootstrap was used to obtain the confidence interval and p-value for the Spearman correlation between the CCFD% and hyperTD area across visits.^40^^–^^42^

All statistical analyses were performed using R version 4.5.1 with the boot, boot.pval, emmeans, geepack, and tidyverse packages.^43^^–^^46^ A two-sided *P*-value < 0.05 was considered statistically significant.

## Results

### Patient and Eye Characteristics

Twenty eyes of 17 patients were included in this study of CCFD% measurements within hyperTDs using SS-OCTA imaging. Each eye was analyzed longitudinally over six visits separated by standard intervals of 12 ± 3 months with an average follow-up time of 59.3 ± 3.1 months (range 54–66 months). The mean age of the 17 patients at baseline was 75 ± 8 years (range 60–87 years) and 13 (76.5%) were women. All eyes had nonexudative AMD throughout the follow-up interval, and three of them started intravitreal pegcetacoplan injections after visit 4, whereas five started intravitreal pegcetacoplan injections after visit 5.

Figure 1 shows an example of the six-visit follow-up series, where all cases had at least one large hyperTD at the first visit, defined by a greatest linear dimension (GLD) of 250 µm on the en face subRPE slab. The hyperTD area at the baseline visit may differ between cases, with some eyes presenting with more advanced atrophy from the onset. By visit 6, all eyes had a large hyperTD that could accomodate a grid box (0.81 mm^2^), ensuring that at that final visit, the target box was always measuring the CCFD% in a region entirely occupied by atrophy as shown in Figure 1F1.

**Figure 2.**
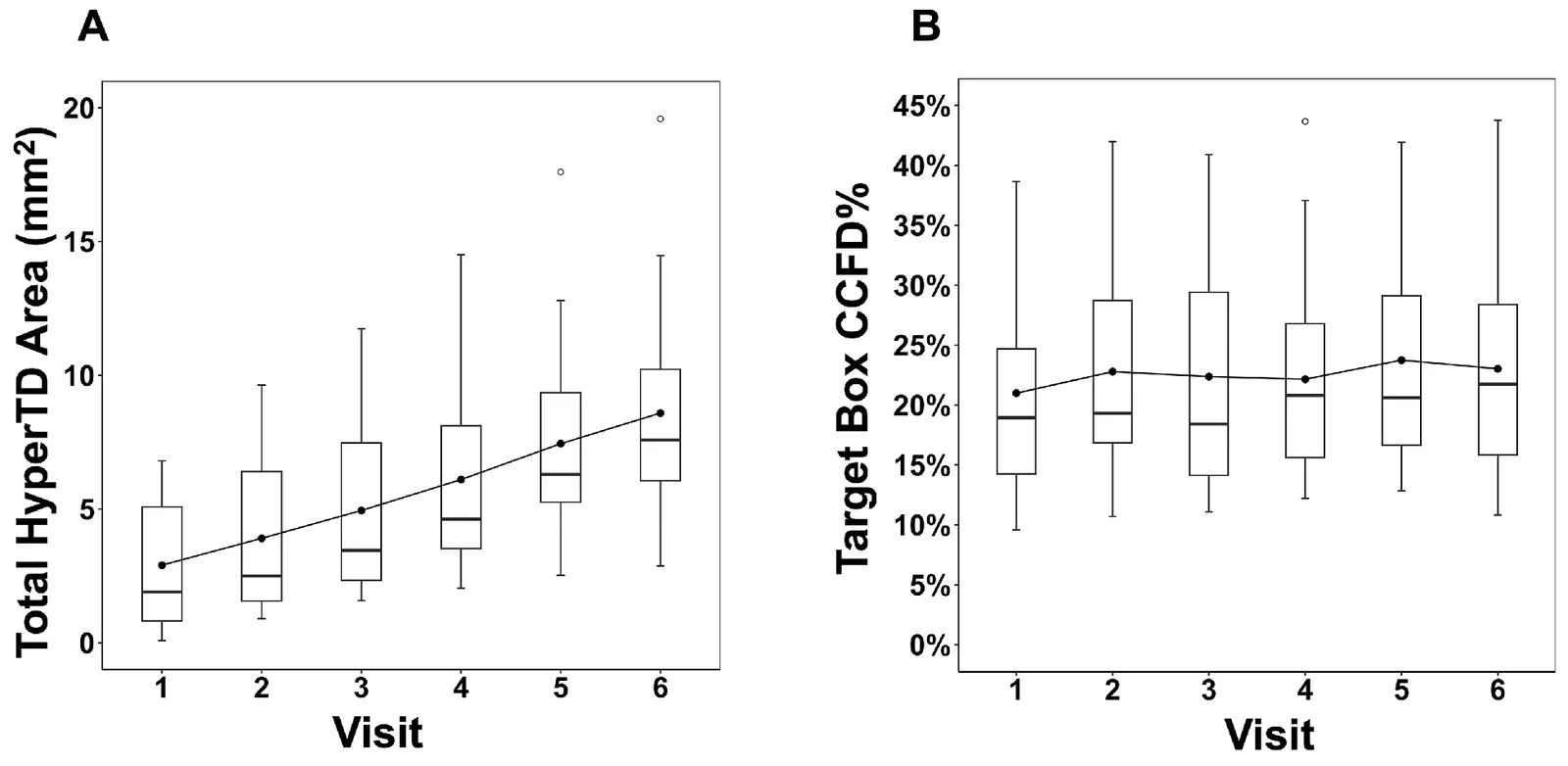
Box-and-whiskers plots of the area of choroidal hyperTDs (**A**) and the target box CCFD% (**B**), which represent the CCFD% at the center of atrophy. Each box shows the interquartile range (IQR), the solid circle within each *box* represents the mean, the *horizontal line* shows the median, the whiskers extend to ± 1.5 × IQR, whereas the *small circles* represent outlier points.

Table 1 shows the mean total hyperTD area of the 20 eyes over the six visits and the estimated mean change from the first visit to each subsequent visit. We note that the area of atrophy progressively increased from visit 1 to visit 6 with an average size of 2.90 mm^2^ at visit-1 to 8.59 mm^2^ by visit 6 (*P* < 0.001). At the end of the follow-up intervals, the areas of the hyperTDs ranged from 2.87 mm^2^ to 19.58 mm^2^. Figure 2A shows the steady growth over time of the mean hyperTD area across 20 eyes in a box-and-whiskers plot.

**Table 1.** Total hyperTD Area Averaged Across 20 Eyes and EMC of Each Follow-Up Visit Compared to the Baseline Visit

Visit	Total HyperTD Area (mm^2^), Mean (SD) [Min-Max]	EMC (95% CI)	*P*-Value
1	2.90 (2.47) [0.08–6.80]	NA	NA
2	3.91 (2.84) [0.91–9.63]	1.01 (0.60 to 1.41)	<0.001
3	4.95 (3.21) [1.58–11.74]	2.04 (1.33 to 2.76)	<0.001
4	6.10 (3.45) [2.04–14.51]	3.20 (2.21 to 4.19)	<0.001
5	7.44 (3.86) [2.53–17.60]	4.54 (3.23 to 5.85)	<0.001
6	8.59 (4.25) [2.87–19.58]	5.69 (4.06 to 7.31)	<0.001

EMC, estimated mean change from the first visit; Min, minimum; Max, maximum.

Figures 1A3–F3 demonstrate the placement of the box grid across the binary CCFD maps and the placement of the target box in red. The target boxes were initially positioned at the center of the hyperTD at the final visit, and then all the previous visits were registered to the final visit. Non-target boxes were referred to as background boxes. Across our 20 cases there were 350 grid boxes that overlapped with areas of hyperTDs in at least one of the visits, and these boxes were labeled as background hyperTD boxes. There were 143 grid boxes that remained unaffected by hyperTDs throughout the 6-visits, and these boxes were labeled non-hyperTD boxes. A total of 17 background boxes were excluded according to the 25% threshold criteria for masking as discussed in the Methods section.

### Target Box CCFD% Measurements

Table 2 shows the mean target box CCFD% measurements of the 20 eyes over six visits and the estimated mean change from the first visit for each subsequent visit. The mean target box CCFD% values at subsequent visits did not show a statistically significant change from the first visit (all *P*s ≥ 0.51). Some cases maintained values as low as 10.8% by the final visit, whereas the maximal recorded CCFD% values reached 43.8% displaying what appeared to be a ceiling effect in the measurable CCFD% values. Figure 2B shows the box-and-whiskers representation of these results, indicating minimal changes across visits for the averaged target box CCFD% measurements and a ceiling value for the CCFD% measurements. Additionally, when assessing both Figure 2A and Figure 2B, we observed that CCFD% measurements in the center of atrophy did not progress in synchrony with the advancement of disease as indicated by the overall growth of the hyperTD area. This was also substantiated by the lack of statistical significance for the Spearman correlation between the CCFD% and hyperTD area across visits, *r* = 0.18 (95% confidence interval, −0.09 to 0.45); *P* = 0.19.

**Table 2.** Target Box CCFD% Measurements Averaged Across 20 Eyes and EMC of Each Follow-Up Visit Compared to the Baseline Visit

Visit	CCFD%, Mean (SD) [Min-Max]	EMC (95% CI)	*P*-Value
1	20.99% (8.33%) [9.57%–38.65%]	NA	NA
2	22.79% (9.19%) [10.70%–41.97%]	1.80% (−1.86% to 5.46%)	0.78
3	22.37% (9.55%) [11.09%–40.90%]	1.38% (−2.21% to 4.98%)	0.93
4	22.15% (8.56%) [12.21%–43.67%]	1.16% (−2.16% to 4.49%)	0.96
5	23.75% (9.37%) [12.85%–41.93%]	2.76% (−1.68% to 7.19%)	0.51
6	23.02% (8.73%) [10.83%–43.76%]	2.03% (−2.76% to 6.83%)	0.88

### Background Box CCFD% Measurements

**Figure 3.**
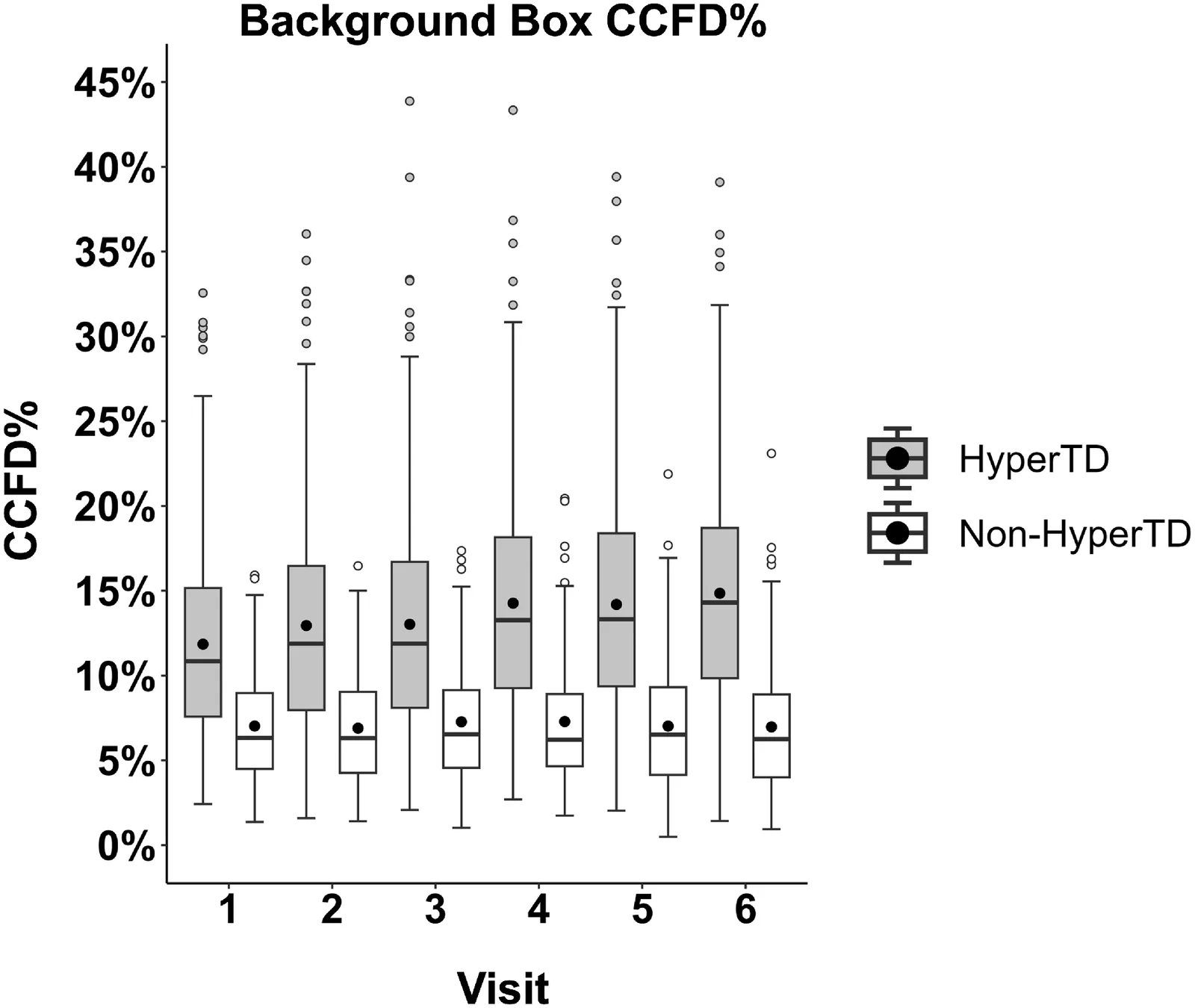
Box-and-whiskers plot of CCFD% in non-target background boxes across 20 AMD eyes. Each *box* shows the interquartile range (IQR), the *solid circle* within each box represents the mean, the horizontal line shows the median, the whiskers extend to ± 1.5 × IQR, whereas the *small circles* represent outlier points. The background boxes are defined as any non-target grid box. They are divided into hyperTD boxes (350 boxes, shown in *gray*), defined as any box that overlapped with areas of hyperTDs in at least one of the visits and non-hyperTD boxes (143 boxes, shown in *white*), defined as any box that remained unaffected by hyperTDs throughout the six visits.

We defined background boxes as any non-target boxes, and these were categorized as hyperTD-boxes, if the box location overlapped with any region of a large hyperTD at any visit, and non-hyperTD-boxes, if the box location remained unaffected by hyper-TDs throughout the follow-up interval. Table 3A shows the mean CCFD% measurements for all background boxes of the 20 eyes in our sample and the estimated mean difference between hyperTD and non-hyperTD boxes at each study visit. Table 3B shows the estimated mean change from the first visit to each subsequent visit for hyperTD and non-hyperTD boxes. For each visit, there was a statistically significant difference between hyperTD boxes and non-hyperTD boxes, with hyperTD-boxes consistently showing higher CCFD% results (all *P*-values < 0.001). The change from the first visit was statistically significant for each subsequent visit for hyperTD boxes (all *P*-values < 0.001) but not for non-hyperTD boxes. Figure 3 shows these results in a box-and-whiskers plot, where hyperTD-boxes shown in gray displayed higher values and wider ranges than non-hyperTD-boxes shown in white.

**Table 3A.** Background Box CCFD% Measurements for HyperTD and Non-HyperTD Boxes Averaged Across 20 Eyes and EMD at Each Study Visit

	CCFD%, Mean (SD) [Min-Max]			
Visit	HyperTD	Non-HyperTD	EMD (95% CI)	*P*-Value
1	11.85% (5.91%) [2.42%–32.56%]	7.02% (3.27%) [1.37%–15.92%]	4.83% (3.09% to 6.56%)	<0.001
2	12.95% (6.38%) [1.59%–36.04%]	6.90% (3.46%) [1.40%–16.47%]	6.05% (4.00% to 8.11%)	<0.001
3	13.02% (6.50%) [2.08%–43.87%]	7.27% (3.52%) [1.03%–17.36%]	5.75% (3.58% to 7.92%)	<0.001
4	14.27% (6.73%) [2.70%–43.34%]	7.29% (3.85%) [1.74%–20.44%]	6.98% (4.63% to 9.33%)	<0.001
5	14.20% (6.65%) [2.04%–39.41%]	7.02% (3.84%) [0.49%–21.89%]	7.18% (4.85% to 9.50%)	<0.001
6	14.85% (6.81%) [1.42%–39.09%]	6.97% (3.85%) [0.94%–23.10%]	7.88% (5.53% to 10.23%)	<0.001

**Table 3B.** EMC for Background Box CCFD% Measurements for HyperTD and Non-HyperTD Boxes of Each Follow-Up Visit Compared to the Baseline Visit

	CCFD%, HyperTD Boxes		CCFD%, Non-HyperTD Boxes	
Visit	EMC (95% CI)	*P*-Value	EMC (95% CI)	*P*-Value
1	NA	NA	NA	NA
2	1.10% (0.55% to 1.65%)	<0.001	−0.13% (−1.08% to 0.82%)	1.00
3	1.18% (0.38% to 1.97%)	<0.001	0.25% (−0.60% to 1.10%)	0.99
4	2.42% (1.32% to 3.52%)	<0.001	0.26% (−1.59% to 2.12%)	1.00
5	2.35% (1.31% to 3.38%)	<0.001	0.00% (−1.38% to 1.37%)	1.00
6	3.00% (2.04% to 3.96%)	<0.001	−0.05% (−1.62% to 1.51%)	1.00

## Discussion

The usefulness of CCFDs as a biomarker for predicting the progression of GA has been a key area of focus in AMD research. Beqiri et al.^22^ investigated eyes with intermediate dry AMD prior to and immediately after the development of early atrophy, reporting that the loss of CC was observed only after the onset of atrophy. However, once GA is established, Thulliez et al.^16^ revealed a correlation between CCFD% in the regions surrounding GA and the annual enlargement rates of these lesions, a finding also reproduced by Nassisi et al.^17^ Furthermore, Shi et al.^18^ showed that the CCFD% throughout the entire scan region outside of GA, which represented a global decrease in macular perfusion, was predictive of the lesions’ growth rates. Nevertheless, because of the challenges associated with CC segmentation and quantification within the area of established GA, there is a scarcity of reports on the CC findings within this specific region as opposed to peri-lesional and global macular changes.

In this work, SS-OCTA imaging was used to analyze the CCFD% measurements in 20 eyes with late-stage nonexudative AMD defined by the presence of GA, also referred to as large hyperTDs. We performed a localized analysis that focused on the center of atrophy to determine the CCFD% measurements in the center of well-established GA, and then we looked retrospectively in time across six longitudinal visits over five years to assess possible changes in the CCFD% measurements at that fixed location. Our data showed that there were no significant differences in the average CCFD% values at the target box location over time, despite progression of the GA as evidenced by the growth in GA area over time. By the final visit, target box CCFD% values ranged from as low as 10.8% to a maximum of 43.8% across the 20 eyes, all of which had developed an area of atrophy at least large enough to contain and surround the entire target box. Importantly, the observed upper limit for the measured CCFD% in areas of atrophy was lower than expected because total CC loss was expected within GA, and we found that target box CCFD% values remained below 43.8% throughout the follow-up.

Our second analysis focused on background non-target boxes, comparing all boxes that intersected regions of hyperTDs in at least one of the visits to those that were clear of hyperTDs throughout the entire follow-up. We observed statistically significant differences in mean CCFD% measurements between the hyperTD and non-hyperTD background boxes of the 20 eyes at each visit, with the hyperTD boxes consistently showing higher values. We also observed a statistically significant increase in mean CCFD% measurements for the hyperTD background boxes but not for the non-hyperTD background boxes. This is consistent with previous reports showing that CCFD% measurements around an established hyperTDs can be associated with its rate of progression.^16^^,^^18^ Our observations are also consistent with the proposal that the appearance and growth of large hyperTDs may involve separate steps with CC loss occurring after the onset of large hyperTDs and the increase in CCFDs playing an important role in the growth of large hyperTDs or GA. In addition, we found a maximum CCFD% measurement of 39.09% in background hyperTD boxes by the final visit, which was consistent with the apparent ceiling effect observed in the target boxes.

**Figure 4.**
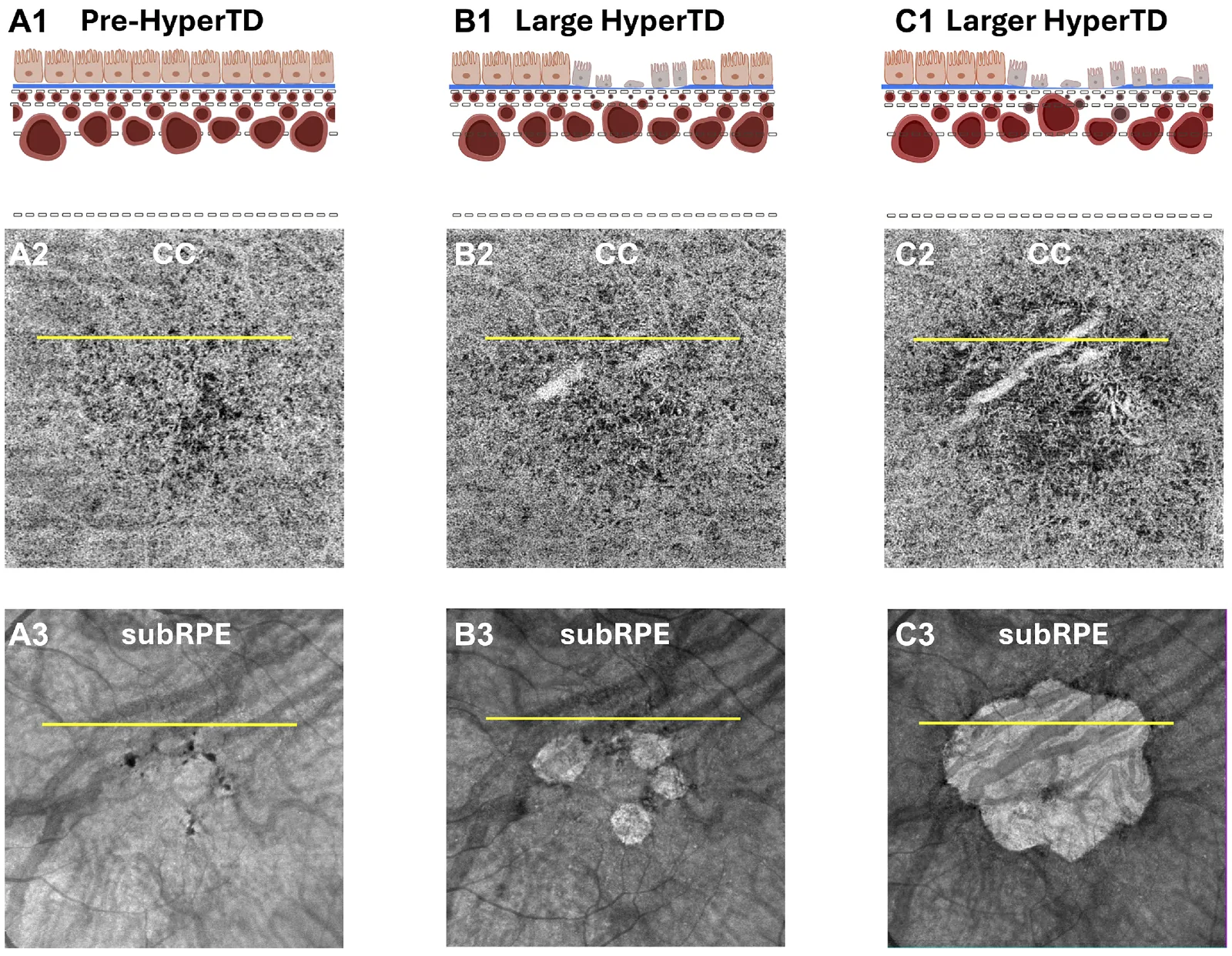
Schematic representation of an eye with AMD showing the anterior displacement of the larger choroidal vessels as the choriocapillaris is lost within the large hyperTD. **(A1–C1)** A schematic depiction of the cross-section of an eye corresponding to the location of the *yellow line* in **A2–C2** and **A3–C3**. The RPE is represented by a single row of polygonal cells, whereas the BM is represented by the blue line underlying the RPE cells. The first two *white dashed lines* are located at 4 µm to 20 µm under the BM and bound the location of the CC, depicted as the smaller circular vessels. The choroidal vasculature, located beneath the CC is represented by intermediate and large vessels. The boundaries of the subRPE layer are indicated by the third and fourth white dashed lines located at 64 µm to 400 µm under the BM. **(A2–C2)** The en face angiographic CC SS-OCTA images segmented at 4 µm to 20 µm under the BM, with darker areas indicating CC attenuation and larger caliber white vessels indicating the signal of blood flow in the choroidal vasculature. **(A3–C3)** The en face subRPE slab segmented at 64 µm to 400 µm under the BM. The *yellow lines* in **A2–C2** and **A3–C3** indicate the position of the cross-sectional diagrams from **A1–C1**. **(A1–A3)** The pre-hyperTD stage. With the onset of large hyperTDs **(B1–B3)**, we see CC impairment and rising choroidal vessels from the underlying Haller's and Sattler's layers. **(B1)** A representative cross-sectional diagram with RPE cells showing focal damage and attenuated CC vessels. A later timepoint **(C1–C3)** shows progression of the hyperTD area, extensive CC damage and prominent choroidal vessels. The depiction in **C1** shows damaged RPE cells overlying a severely attenuated CC layer, and choroidal vessels markedly shift superiorly. Created in BioRender. Beqiri, S. (2026) https://BioRender.com/y65oovk; https://BioRender.com/rlumpzn; https://BioRender.com/57em4yv.

Our results suggest that we were unable to measure the complete loss of CC within atrophy, presumably because the space normally occupied by the CC is replaced by the larger vessels from the Sattler and Haller choroidal layers as the CC perfusion decreases and vessels are lost (Fig. 4). These findings are consistent with the results reported by Moult and colleagues.^47^ Using SS-OCTA to image the CC under nascent GA and drusen-associated GA, they found that the CC angiographic signal remains present due to the displacement of the underlying choroidal vasculature towards BM. Nesper et al.^48^ reported similar observations when medium sized choroidal vessels were anteriorly displaced across all studied eyes within GA and were visually overlapping the CC slab. Although they used an SD-OCTA device and applied a wider and deeper slab than in our investigation, their observations were confirmed using histology, demonstrating choroidal vessel abutting the BM, separated by only a narrow 1–2 µm distance, a markedly reduced distance when compared with healthy tissue.

**Figure 5.**
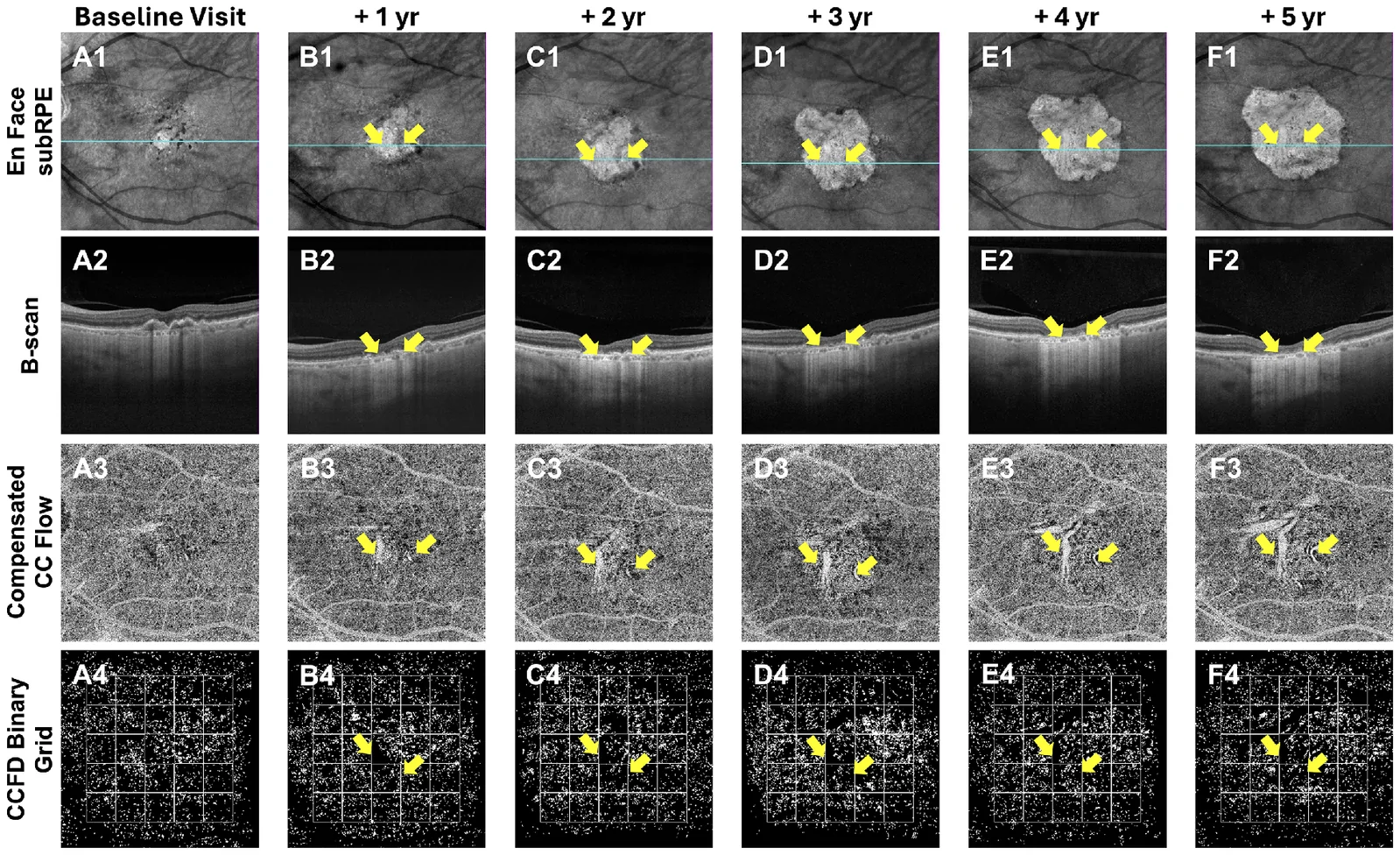
Example of an eye with AMD showing the progression of choroidal hyperTD and the quantification of flow in the CC slab. **(A1–F1)** The en face SS-OCTA subRPE slab across six visits over five years. HyperTDs are seen as bright areas on the subRPE slab. The *blue line* displays the location of the B-scans shown in **A2–F2**. The *yellow arrows* on the en face subRPE view correspond to the *yellow arrows* in the B-scans pointing at two large choroidal vessels abutting the Bruch's membrane within the region of atrophy. **(A3–F3)** The compensated CC flow images. Loss of CC corresponds to dark areas on the en face images, and the underlying choroidal vessels indicated by the *yellow arrows* become more visible, corresponding to the same arrows from the en face subRPE and B-scan views. **(A4–F4)** The binary CCFD grid maps, obtained by thresholding and registering the images in **A3–F3**. *White areas* on the binary maps represent flow deficits, whereas black regions represent presence of flow. All binary maps are registered to the final visit and the superimposed grid is used to track the CCFDs measurement from the same regions over time. The *yellow arrows* indicate the location of the same two choroidal vessels that appear as black in the binary map and are recorded as flow within the CC slab.

In Figures 1 and 5, we demonstrated the prominence of the choroidal vessels in the en face CC slab at the area of atrophy. This anterior displacement is schematically shown in Figure 4, where we proposed that the degenerative changes in advanced AMD present a complex dynamic picture with the loss of CC structure accompanied by an anterior shift of medium sized choroidal vessels from the underlying Haller and Sattler layers. Our quantitative analysis of the CCFD% measurements in GA showed that detectable CCFD% values did not exceed the low forties. We must emphasize that this observed plateau does not indicate a stabilization of CC perfusion, but more likely reflects our inability to detect the decreasing CC perfusion values due to the anterior displacement of both Sattler's and Haller's layers resulting from the loss of the CC and the loss of the anatomic space previously occupied by the CC (Figs. 1, 4, 5). Combining the qualitative and quantitative observations, we propose that this ceiling effect is a manifestation of the flow signals from choroidal vessels replacing the CC flow signal resulting in the inability to show more extensive CC loss using our current methodology. Therefore our primary aim is to advise caution when assessing the CC within areas of GA or areas evolving toward GA.

Identifying this ceiling effect with respect to the detection of CCFD% calls into question several studies that report values above 50%.^49^^–^^52^ Usually these values are detected under drusen, and the most likely explanation for these higher values of CCFDs is that the authors failed to adequately compensate these scans. As a result, they did not accurately detect the CC perfusion under drusen due to the attenuation of the signal, especially when using spectral-domain OCT angiography.^50^^–^^55^ By using SS-OCTA and our compensation strategy, we have shown that CCFDs are not typically increased under drusen and only increase after the formation of hyperTDs.^21^^,^^22^^,^^28^^,^^56^

Key strengths of our methods include the use of a well-developed quantitative strategy for measuring CCFD% values from a CC slab that is correctly positioned anatomically. Our CC visualization and quantification technique is based on the most up-to-date guidelines, selecting an anatomically correct CC slab location, manually checking for CC segmentation errors, minimizing sources of artifacts such as CaD and HRF, optimizing the compensation level and adjusting it for areas of hyperTD, as well as implementing a validated thresholding strategy.^25^^,^^27^^,^^28^ Second, our SS-OCTA device with a 1050 nm wavelength allowed deeper light penetration and lower signal roll-off into the CC and choroid compared with the alternative SD-OCTA device with a lower wavelength of 840 nm and poorer penetration through the RPE and drusen.^14^^,^^57^ Additionally, our dataset consisted of six longitudinal visits over five years for each of the 20 eyes and provides robust evidence for our observed plateau in CCFD% measurements. Last, our grid strategy facilitates correlation of structural changes in the RPE to functional changes in CC perfusion, allowing a focused analysis of the target area in the center of atrophy, as well as a secondary investigation of all hyperTD-affected and unaffected boxes.^21^

A limitation of our method is the semi-automated nature of the CC segmentation algorithm. Because of the challenges of the RPE-BM complex anatomy in eyes with atrophy, this segmentation process required significant manual labor and oversight. However, given the very narrow depth of the CC slab, as well as the challenging anatomy within the area of GA, there may have been subtle segmentation artefacts that escaped the evaluation by the grader, but these artefacts would have had minimal impact on the results. Similarly, we excluded the possibility of projection artefacts from the retinal circulation due to improved signal penetration into the choroid when the overlying RPE and CC are lost or attenuated. There were no retinal vessels overlying the CC maps that could account for the larger vessels observed.

Our focus on a single central target box also presents another limitation, as we may not fully capture the intralesional heterogeneity of CC. Nevertheless, this strategy allowed us to explore longitudinal changes within a specific area of GA across all visits, whereas our secondary analysis comparing all hyperTD grid boxes to all those not affected by hyperTD provided a more generalizable summary of changes occurring in non-target boxes and revealed similar findings of a plateau in the recordable CCFD% values.

Additionally, in this current study we have not characterized the features of the choroid. Shi et al.^18^ demonstrated that choroidal vascular index inside the GA was indicative of future growth of GA. Future investigation will focus on quantifying the changes in the choroid, and specifically the anterior shift observed in the area of atrophy.

The fact that eight of the 20 eyes received pegcetacoplan injections because of the extent of atrophy is a potential confounding factor; however, given that there is no evidence that pegcetacoplan should affect CC perfusion, we do not believe that this intervention had a meaningful impact on our observations. Furthermore, we repeated our analysis and excluded the pegcetacoplan-injected eyes. We found the same findings for the remaining treatment-naïve eyes, with no significant changes in target box CCFD% across visits (all *P* ≥ 0.08). Moreover, the changes from baseline in total hyperTD area at each subsequent visit from baseline were statistically significant (all *P* < 0.001), consistent with the observations from the complete cohort. Nevertheless, we are currently investigating if pegcetacoplan has any impact on the choroidal vasculature.

Last, we must emphasize that CCFD% is a binary metric resulting from a thresholding process, below which we consider the perfusion as undetectable. However, the ability to detect perfusion depends on the imaging protocol, scanning speeds, signal processing algorithms and workflow used for quantification.^25^^,^^26^ Other approaches, such as using the Variable Interscan Time Analysis, may provide information on CC flow velocity that may be able to differentiate more precisely between an angiographic signal arising from the CC or choroidal vessels.^29^

## Conclusions

Our study demonstrates a model of dynamic structural changes within the CC and choroidal vasculature associated with GA. The observed ceiling effect suggests that a maximum CCFD% measurement value well below 50% is obtained within areas of atrophy because the angiographic signal arises from the displaced choroidal vasculature. Hence, any values of CCFD% greater than 50% should be viewed cautiously and may reflect errors in the detection or processing of CCFD% measurements.
